# Fine-mapping of microbeam radiation therapy-induced DNA damage in 2D cell and 3D epidermis models

**DOI:** 10.1186/s13014-026-02952-4

**Published:** 2026-10-26

**Authors:** Mai Nguyen, Annique C. Dombrowsky, Franziska Treibel, Jessica Müller, Susanne Raulefs, Stefan Bartzsch, Stephanie E. Combs, Stefan Eder, Thomas E. Schmid, Harry Scherthan

**Affiliations:** 1 TUM School of Medicine and Health, Department of Radiation Oncology, TUM University hospital, Technical University of Munich, Munich, Germany; 2 German Research Center for Environmental Health, Institute of Radiation Medicine, Helmholtz Zentrum München GmbH, Neuherberg, Germany; 3 Bundeswehr Institute of Radiobiology affiliated to the University of Ulm, Neuherbergstr. 11, 80937 Munich, Germany

**Keywords:** Microbeam radiation therapy, Biological dosimetry, CHO-K1 cells, Skin tissue, Micronuclei, Nucleoplasmic bridges, 53BP1, yH2AX

## Abstract

**Background:**

Microbeam radiation therapy (MRT) delivers spatially fractionated, high-dose radiation in parallel beam arrays, creating spatial fractionation by alternating peak and valley dose regions. While this approach offers significant potential for tumor control with reduced normal tissue toxicity, the precise biological dose and damage distribution, particularly the relative contributions of direct and non-targeted (bystander) effects, remain incompletely understood.

**Methods:**

We employed complementary biodosimetric approaches to quantify DNA damage responses in two-dimensional CHO-K1 cell monolayers and three-dimensional epidermis tissue models following MRT. Biological dosimetry in CHO-K1 monolayers was performed using the cytokinesis-block micronucleus (CBMN) assay for micronuclei (MN) and nucleoplasmic bridges (NPB). In epidermis models, DNA double-strand break (DSB) damage was analyzed by immunofluorescence (IF) with γH2AX+53BP1 DSB markers. Spatial and temporal mapping of damage in tissue sections was performed across microbeam peak and valley regions and compared with physical dosimetry and broadbeam irradiation.

**Results:**

In 2D CHO-K1 cells, MN- and NPB-based biological dosimetry closely matched physical dose measurements, with accurate dose reconstruction in valleys but substantial underestimation in peaks due to extensive cell death and loss of scorable nuclei. In 3D epidermis models, MRT induced distinct spatial patterns of DNA damage with pan-γH2AX–positive cells indicating excessive DSB formation at peak regions at early time points, whereas 53BP1 DSB foci predominated in dose valleys. Over 24 h, pan-γH2AX signals declined to sham levels, while 53BP1 foci persisted, particularly in peak regions, indicating DNA damage regions refractory to repair. Comparative analyses revealed that valley cells exposed to 1 Gy MRT radiation exhibited in average significantly more 53BP1 foci at 0.5 h than after uniform 1 Gy broadbeam irradiation, suggesting a bystander contribution from adjacent high-dose peaks. By 24 h, however, damage levels converged between MRT valleys and broadbeam, indicating that bystander-associated excess damage was transient.

**Conclusion:**

CBMN-based biological dosimetry reliably quantifies absorbed dose in MRT valleys but is limited in peak regions. 3D epidermis models enable spatial and temporal mapping of DNA damage, revealing persistent complex damage in peak-region cells and provide evidence for transient bystander effects in valley regions. Extending previously established biodosimetric and DNA damage mapping approaches to X-ray MRT, these findings, obtained in one cell line and one tissue model, contribute to the understanding of DSB induction, repair dynamics, and non-targeted effects in MRT.

## Introduction

Microbeam Radiation Therapy is an experimental technique in radiation oncology that aims to enhance therapeutic efficacy while minimizing side effects. It uses a collimator to spatially modulate the radiation dose into arrays of planar microbeams, each with a width down to tens of micrometers and peak doses reaching hundreds of Gray [32]. These high-dose “peaks” are separated by low-dose “valley” regions receiving radiation below normal tissue tolerance, thereby sparing surrounding healthy tissue more effectively than conventional radiotherapy [23, 31, 33]. This spatial fractionation concept underlies the fundamental therapeutic advantage of MRT: the ability of normal tissues to withstand extreme peak doses as long as sufficient low-dose regions are preserved to sustain tissue integrity and regenerative capacity.

Despite its promise, MRT remains technically challenging. Efforts to develop compact MRT systems include approaches based on inverse Compton scattering [20], carbon nanotube sources [29], and modified X-ray tubes [5, 11].

Effective microbeam generation requires low beam divergence, a small source size, and photon energies around 100 keV to maintain beam shape at increasing tissue depths. High dose rates and short exposure times are also critical to prevent motion-induced blurring of the micrometer-scale dose patterns. Currently, only large third-generation synchrotrons provide suitable beam properties, limiting MRT’s development over the past 25 years to a limited number of facilities [30]. This dependence on synchrotron infrastructure has significantly constrained systematic radiobiological research, hindering progress toward clinical translation and leaving fundamental biological questions unresolved.

Preclinical studies have demonstrated successful tumor ablation in mice and rats using MRT (Laissue et al., 1998 [14]), . Although the radiobiological mechanisms remain unclear, evidence suggests that the juxtaposition of high and low dose regions may trigger an immune response [21, 35]. Additional findings indicate that MRT may modulate vascular integrity, alter intercellular signaling, and induce non-targeted effects, all of which could contribute to its unusually high normal tissue tolerance and tumor control potential. Nonetheless, the mechanistic interplay between physical dose deposition, DNA damage response, and tissue-level outcomes remains poorly characterized [16].

Previously, we used microbeams of 50 μm width with an X-ray tube source (established by Bartzsch et al. [5]) in vitro experiments, achieving dose rates of ≤ 18 Gy/min. The current version of our small-animal irradiator generates microbeams with adjustable widths (~ 20–100 μm) and dose rates up to 8.0 Gy/min (see Fig. 3 in [36]). Using a collimator with variable and tilted slits mounted at 21.2 cm source distance, the system is optimized for dose rate, peak-to-valley dose ratio (PVDR), and field homogeneity.

Physical dosimetry was validated biologically via a cytokinesis-block micronucleus (MN) assay, which showed dose-dependent increases in micronuclei formation in MRT peak regions across 2D cell cultures [36]. Until now, further biological endpoints and the underlying DNA damage contributing to MN formation have not been explored. In particular, it remains unknown how spatial dose modulation influences chromosomal instability, DNA repair kinetics, and non-targeted effects within peak and valley regions.

In our current study, we expanded the MN biodosimetry analysis to include nucleoplasmic bridges (NPBs), structures linked to chromosomal instability. NPBs form when a chromatid connects two daughter nuclei containing a dicentric chromosome induced by misrepair or telomere fusions (for a review see [13]) and are expected to appear at higher frequencies under high-dose irradiation such as MRT.

Although the cytokinesis-block micronucleus (CBMN) assay provides a fast and accurate method for quantifying biological dose after MRT in 2D cell culture systems [36], its applicability is limited by the lack of 3D tissue architecture and the absence of physiologically relevant cell–cell and cell–matrix interactions [3]. However, such structural and microenvironmental features are crucial for capturing non-targeted responses, such as radiation-induced bystander effects [25], which cannot, due to absence of 3D tissue geometry and specific cell-cell contacts, be fully represented in monolayer cultures. Consequently, a more physiologically relevant model is needed to investigate these effects under conditions that more closely mimic in vivo tissue organization. Therefore, we also investigated MRT-induced dsDNA damage in a 3D epidermis tissue model using a high-resolution spatial DSB damage mapping strategy based on γH2AX and 53BP1 DSB damage foci, previously developed for proton mini-beam radiotherapy [27].

Building on our previous studies of X-ray microbeam biodosimetry in CHO-K1 cells [36] and proton minibeam-induced DNA damage in reconstructed epidermis models [27], the present study addresses several previously unresolved questions. First, we extend biological dosimetry by introducing nucleoplasmic bridges (NPBs) as a complementary cytogenetic endpoint and evaluate their performance relative to micronuclei for biological dose reconstruction in peak and valley regions. Second, we establish high-resolution spatial mapping of DNA damage after X-ray microbeam radiation therapy (MRT) in a physiologically relevant human 3D epidermis model. Finally, by comparing MRT valley regions with homogeneous broadbeam irradiation delivering the same valley dose, we investigate the contribution of transient bystander responses to DNA damage induction. In summary, our analyses allowed us to dissect the spatial, temporal, and mechanistic dimensions of MRT-induced DNA damage and bystander effects. Methodologically, the present work should therefore be regarded as an extension of these established approaches to X-ray MRT rather than as a new methodology. Beyond the points outlined above, direct biodosimetric measurements after MRT with a valley dose of 2 Gy allowed us to define the distance from the peak center below which cytogenetic reconstruction of the valley dose becomes unreliable.

## Methods

### Irradiation

Irradiation of reconstructed skin tissues was performed using the microbeam collimator mounted on a XenX small-animal irradiator (Xstrahl, Camberley, UK). To ensure accurate dose delivery, film dosimetry was conducted prior to tissue exposure. For this purpose, a piece of Gafchromic™ EBT3 film was positioned between a 2 mm and a 9 mm PMMA plate to replicate the geometry of the skin tissues within their culture inserts and irradiated under identical conditions. The resulting dose rates were determined as 4.1 ± 0.2 Gy/min in the microbeam peak and 0.112 Gy/min in the valley regions. For comparison, broadbeam irradiation was performed at a X-Strahl RS225 system (Xstrahl Ltd, Camberley, UK), which yielded a dose rate of 7.06 ± 0.02 Gy/min. Furthermore, the peak-to-valley dose ratio (PVDR) was measured with film dosimetry (Gafchromic EBT3 films) and microscope read-out. In short, films were calibrated between 1 and 6 Gy. MRT profiles were acquired by a peak and a valley exposure, where peak and valley dose were in the range between 1 and 6 Gy. Films were scanned with an Axio Imager Z1 microscope (Zeiss, Oberkochen, Germany). Details are described in [36] and [4].

To replicate the irradiation and dosimetry setup as closely as possible while maintaining sterility, the epidermis models were transferred from their inserts into 35 × 10 mm Nunc™ cell culture Petri dishes (Thermo Fisher Scientific, Massachusetts, USA). Each dish was filled to the brim with PBS to avoid air pockets and sealed with SealPlate^®^ film (Excel Scientific, Victorville, CA, USA). To facilitate orientation during irradiation, two opposing holes were introduced into each sample using a 20G needle (Sterican, Braun, Melsungen, Germany). The axis of these holes was marked on the SealPlate^®^ film to ensure alignment of the tissues parallel to the microbeam orientation during exposure.

Irradiation was carried out with the XenX system operated at 225 kV and 13 mA using a large focal spot. A slit width of 50 μm was applied, with a copper filter in place. The center-to-center (ctc) beam spacing was set to 400 μm. During irradiation, the tissue samples were positioned directly on the collimator and exposed from below.

To achieve a peak dose of 19 Gy and a valley dose of 1 Gy tissues were first exposed to microbeam irradiation and subsequently to broadbeam irradiation. For comparison, a reference broadbeam irradiation delivering a homogeneous 1 Gy dose was performed using the RS225 irradiator operated at 220 kV and 1 mA with the same copper filter configuration as in the XenX system. This allowed direct comparison between heterogeneous microbeam irradiation and uniform broadbeam exposure at equivalent valley doses.

### Biodosimetry MN and NPB analysis in 2D CHO-K1 cell cultures

To extend the micronucleus biodosimetry in microbeam radiation therapy reported by Treibel et al., [36], we re-analyzed micronucleus (MN) formation in CHO-K1 cells (Chinese hamster ovary cells; German Collection of Microorganisms and Cell Cultures (DSMZ), Braunschweig, Germany) under 2D culture conditions. CHO-K1 cells were irradiated at approx. 60% confluency and fixed 27 h after irradiation. Immediately following irradiation (24 h after seeding), Cytochalasin B was added to block cytokinesis. In addition, we evaluated NPBs as a complementary endpoint, which were not addressed in the original study. In our analysis, nucleoplasmic bridges were characterized by a variable width not exceeding one-quarter of the nuclear diameter, following the scoring criteria described by Fenech et al., [12].

Importantly, MN and NPBs were only assessed in 2D cell cultures, since reliable scoring in 3D tissue models was not feasible. This drawback was resolved by studying DNA in 3D epidermis models (see below).

### 3D Human epidermis model

We employed Phenion^®^ epiCS reconstructed human epidermis models (CellSystems Troisdorf, Germany; now Fraunhofer TLZ-RT, Würzburg, Germany). Each tissue model has a surface area of 0.6 cm² and is reconstructed from primary human epidermal keratinocytes cultured on a polycarbonate filter under air–liquid interface (ALI) conditions. The epidermis models consist of a multilayered, cornified epidermis with a basal cell layer, proliferating suprabasal keratinocytes, and a differentiated stratum corneum that closely mimics native epidermis of human skin. epiCS models have proven suitable for studying proton minibeam radiation-induced DNA damage in a 3D tissue context [27].

Upon arrival, the reconstructed epidermis tissues were handled according to the manufacturer’s recommendations. Briefly, 1 mL of cold epiCS culture medium was added to each well of a 6-well plate. Using sterile forceps, the tissues were carefully transferred into the wells and incubated under standard cell culture conditions (37 °C, 5% CO₂) for 24 h to allow equilibration prior to irradiation. Subsequent processing steps, including fixation and embedding, immunohistochemistry, and microscopic evaluation, followed previously described protocols [27]. These procedures ensured the preservation of tissue architecture and allowed for reliable visualization and quantification of DNA damage and repair markers according to irradiation geometry in the 3D epidermis model.

### Fixation and embedding

Following irradiation, the epidermis models were incubated for 0.5 h and 24 h at 37 °C in a humidified atmosphere containing 5% CO_2_ to allow for either immediate or delayed cellular responses. Tissue fixation was subsequently carried out using 4% paraformaldehyde (PFA) in phosphate-buffered saline (PBS). After a 20-minute fixation period at room temperature, the samples were washed twice in PBS (5 min each) to remove excess fixative, followed by an additional wash in PBS supplemented with 0.1% glycine to quench residual aldehyde groups. Fixed samples were stored in PBS at 4 °C until further processing.

For histological analysis, the protocol of Scherthan et al. [27] was followed: epidermis models together with their supporting membranes were carefully removed from their culture inserts using 8 mm sterile biopsy punches (Thermo Fisher Scientific, Massachusetts, USA) and subsequently embedded in low-melting-point agarose. Dehydration was performed using a graded ethanol series in a Leica ASP300S tissue processor (Leica Biosystems, Nußloch, Germany), followed by paraffin infiltration under standard conditions. The processed tissues were then embedded in paraffin blocks using cube-shaped molds. To preserve the spatial relationship between the radiation tracks and the epidermal architecture, the orientation of the microbeam paths was carefully marked prior to embedding. This enabled sectioning to be performed perpendicular to the beam direction, which is essential for accurate spatial analysis of radiation-induced DNA damage. Finally, according to our lab standard, 5 μm paraffin serial sections were prepared using a Leica RM2255 rotary microtome and mounted on Superfrost^®^ Plus slides (Thermo Fisher Scientific), the section thickness being routinely used in our lab and has yielded good results in previous analyses (e.g., Ahmed et al. [2], Scherthan et al. [27]. These sections were subsequently subjected to immunohistochemistry and microscopic evaluation.

### Immunofluorescence and image acquisition and analysis

Paraffin-embedded tissue sections of 5 μm thickness were processed for immunofluorescence staining to visualize radiation-induced γ-H2AX and 53BP1 surrogate DSB damage foci as described previously [27].

Immunofluorescence microscopy and quantitative image analysis of the epidermal tissues were performed using an Axio Observer Z1 fluorescence microscope (Zeiss, Oberkochen, Germany) equipped with the TissueFAXS digital imaging platform and the associated TissueQuest and StrataQuest analysis software packages (TissueGnostics, Vienna, Austria). This integrated system enabled high-throughput acquisition and automated analysis of fluorescence signals across entire tissue sections.

To evaluate the spatial distribution of radiation-induced DSB markers, each cell nucleus was segmented and annotated based on its position along the tissue axis. In curved tissue sections, DAPI-stained nuclei were computationally mapped relative to a defined reference point at the start edge of the epidermis section. This procedure enabled linear two-dimensional reconstruction of the tissue architecture, thereby allowing direct comparison of foci distribution in single cells across differently oriented sections [27]. This analysis was performed for 6–13 tissue sections (in average 2796 cells analyzed per section) for each exposure modality.

The quantification of spatially mapped DNA damage patterns addressing microbeam peak and valley regions, was performed in MATLAB (MathWorks, Natick, MA, USA) using a custom-developed analysis script. For each nucleus, the distance to the nearest microbeam center was calculated, and nuclei were grouped into 25 μm bins to generate distance–response profiles of foci distribution. Because quantitative analysis was performed on 5 μm paraffin sections rather than intact nuclei, the absolute number of detected DSB damage foci per nucleus represents only a fraction of the total nuclear foci. Furthermore, automated image analysis relies on predefined nuclear segmentation and fluorescence intensity thresholds, which influence the absolute foci counts. Consequently, the reported absolute foci numbers are intended only for quantitative comparison between identically processed samples within the present study or with studies employing the same section-based imaging and analysis methodology. Direct comparison of absolute foci counts with studies using different section thicknesses, imaging modalities, or manual whole-nucleus scoring approaches is therefore not appropriate.

For each irradiation modality and fixation time point, three non-irradiated control samples were included to account for baseline DNA damage. Per experimental condition, 2–4 reconstructed epidermis models were irradiated, and from each model, at least three independent paraffin sections were processed and quantitatively analyzed. This ensured robust statistical evaluation of DNA damage patterns across biological and technical replicates.

### Statistics

All quantitative results are reported as mean values ± standard deviation (SD). Statistical analyses were performed using SPSS (IBM, Ehningen, Germany). To determine differences between experimental groups, a two-tailed Student’s t-test was applied. A p-value < 0.05 was considered indicative of statistical significance.

Prior to hypothesis testing, the data were screened for potential outliers using Microsoft Excel (Microsoft Corporation, Redmond, USA). Outliers were identified according to the interquartile range (IQR) method, whereby values lying more than 1.5 × IQR outside the first or third quartile were flagged. Unless otherwise indicated, these outliers were excluded from subsequent statistical evaluation.

## Results

### Biological dosimetry using micronuclei and nucleoplasmic bridges in a 2D CHO cell model

#### Dose-response-curve for biological dosimetry

**Fig. 1:**
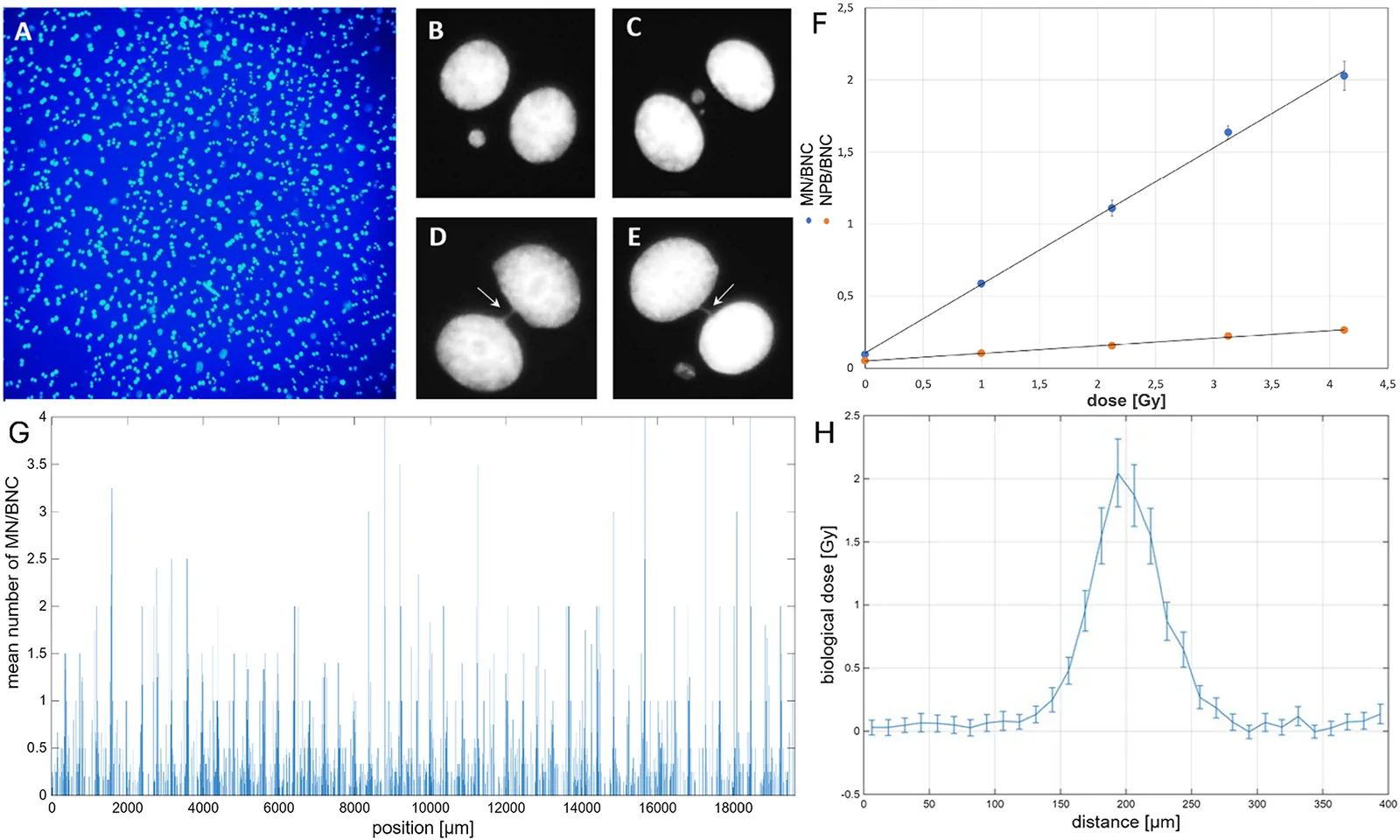
**(A)** Overview of slide used for MN analysis in binucleated cells. **(B-E)** Magnified BN cells showing **(B)** one MN and **(C)** two MN. **(D, E)** BN cells showing nucleoplasmic bridges between daughter nuclei (arrows). **(F)** Dose-response curve for micronuclei and nucleoplasmic bridges in CHO-k1 cells. Average number of micronuclei (MN) per binucleated cell (BNC) (MN/BNC; blue) and average number of nucleoplasmic bridges per binucleated cell (NPB/BNC; orange) induced by 0–4.1 Gy broadbeam irradiation. Each data point represents the mean ± standard deviation; *n* = 3 for all doses except 3 Gy (*n* = 2). The analysis was performed on > 1500 cells per data point. **(G)** Mean number of micronuclei per binucleated cell, averaged over 10 μm bins along the longitudinal axis of an MRT irradiated slide. The MN/BNC peaks correlate with the high dose regions of the planar microbeams. **(H)** Micronuclei biodosimetry based on conversion of average MN frequency/cell-bin to Gy after MRT to CHO-K1 cells with a peak dose of 2 Gy. Biological dose as a function of the distance from the peak center (at ~ 200 μm) following microbeam irradiation. Irradiation was performed with a peak dose of 2 Gy and a PVDR of 40.67. Cells were scored relative to the nearest peak center in 12.5 μm bins across the entire slide, here a 400 μm section of a slide is shown. Each data point represents the mean ± standard deviation; *n* = 3

Micronucleus (MN)-based biological dosimetry is technically challenging in 3D tissue models, as reliable identification and scoring of individual nuclei is impeded by tissue thickness and complex architecture. Therefore, all analyses of MN, nucleoplasmic bridges (NPB) and the establishment of calibration curves were performed exclusively in CHO-K1 cells cultured in 2D on glass slides. This extends the study of Treibel et al., [36], in which MN formation (Fig. 1A–C) was analyzed in the same cell line, by applying an improved scoring strategy and additionally including dose–response calibration curves as well as the quantification of NPB (Fig. 1D, E) as a complementary endpoint of genomic instability.

To enable biological dose estimation, separate dose–response reference curves for MN and NPB were established under identical irradiation conditions. Application of the cytokinesis-block micronucleus (CBMN) assay revealed a linear increase in MN frequency with dose (Fig. 1F). The regression equation for the frequency F of MN in dependence on dose was y = 0.47 D + 0.11 (R² = 0.9985). At 0 Gy, the mean frequency was 0.094 ± 0.009 MN/Binucleated cell (BNC), while at 4 Gy it rose to 2.03 ± 0.10 MN/BNC.

In addition, nucleoplasmic bridges were detected in the same CBMN assay preparations. The NPB calibration curve likewise followed a linear trend (Fig. 1F), described by y = 0.052 D + 0.050 (R² = 0.9959). At baseline, the mean was 0.05 ± 0.01 NPB/BNC, which increased to 0.26 ± 0.02 NPB/BNC at 4 Gy. The slope of the NPB curve was approximately tenfold lower than that of the MN curve (Fig. 1F), indicating that bridges occur at substantially lower frequency than micronuclei under identical conditions.

Taken together, these results demonstrate that in CHO-K1 cells, both MN and NPB can be used as reliable biodosimetric markers. While MN provide a highly sensitive measure of absorbed dose, NPB occur at a markedly lower frequency. The slope of the NPB calibration curve was approximately tenfold lower than that of the MN curve, corresponding to ~ 10% of the MN frequency under identical irradiation conditions. This finding suggests that chromosomal misrepair events leading to bridge formation occur at about one-tenth the rate of missegregation events resulting in micronuclei, thereby adding a quantitative dimension to the interpretation of radiation-induced genomic instability.

#### MN biodosimetry after MRT with a peak dose of 2 Gy in CHO-K1 cells

Following irradiation of a 2D CHO-K1 cell model with MRT [36], distinct clusters of micronuclei were observed at regular intervals along the X-axis coordinates of the irradiated cells, corresponding to the microbeam peaks, with a sharp decrease evident in the valley dose regions (Fig. 1G and H).

To evaluate the absorbed dose distribution following microbeam radiation therapy (MRT), the frequency of radiation-induced chromosomal damage was quantified by scoring the mean number of micronuclei per binucleated (BN) cell and the mean number of nuclear bridges per BN cell. These endpoints were analyzed as a function of the distance from the center of the irradiation peak using MATLAB. More than 1100 cells per data point were analyzed. The biological dose was then derived by converting these values into dose equivalents based on established calibration curves (Fig. 1F).

For microbeam irradiation with a nominal peak dose of 2 Gy and a valley dose of 0.05 Gy, biological dosimetry using the MN assay yielded an estimated biological dose of 1.98 ± 0.18 Gy (corresponding to 1.05 ± 0.09 micronuclei per BN cell) in the peak region, and 0.10 ± 0.05 Gy (0.15 ± 0.03 micronuclei per BN cell) in the valley region (Fig. 1H). These values were in excellent agreement with the corresponding physical dose measurements obtained using Gafchromic film, demonstrating consistency between biological and physical dosimetry approaches.

#### MN biodosimetry after MRT with a valley-dose of 2 Gy

To accurately quantify the valley dose from MN induction in CHO-K1 cells, microbeam photon irradiation was performed with a nominal valley dose of 2 Gy and a corresponding peak dose of 81.34 Gy. More than 1000 cells per data point were analyzed. Biological dosimetry, based on MN frequencies determined in 12.5 μm bins along cross-sectional profiles, yielded an estimated dose of 2.07 ± 0.15 Gy (corresponding to 1.09 ± 0.07 MN per BN cell) at the center of the valleys, located approximately 194 μm from the peak center. At these positions, the measured biological dose was in close agreement with the physical dose obtained from Gafchromic film, confirming the reliability of MN-based biological dosimetry for valley regions.

In contrast, biological dosimetry could not be applied within the high-dose peak regions or within ~ 81 μm from the peak center, as the extremely high peak doses induced extensive DNA damage, resulting in prolonged cell-cycle arrest, mitotic failure and/or reproductive cell death. Consequently, only very few cells completed cytokinesis and fulfilled the scoring criteria for MN and NPB analysis, precluding reliable biological dose reconstruction in the peak regions. The mean spacing between microbeam peaks was 402.5 ± 0.3 μm, which is in good agreement with irradiation geometry.

Given the established relevance of valley dose for normal tissue sparing in MRT [34], the valley region was analyzed in greater detail. Specifically, data obtained under conditions of a 2 Gy valley dose were compared with results from an irradiation scheme delivering a 2 Gy peak dose, corresponding to 0.05 Gy in the valley. For comparison, the 2 Gy peak-dose profile was scaled by a factor of 40.67, corresponding to the peak-to-valley dose ratio (PVDR), and aligned with the experimental data from the 2 Gy valley-dose irradiation.

This analysis revealed that irradiation with 2 Gy in the valley provided a more accurate and direct characterization of the valley dose distribution compared to dose reconstruction via scaling of the peak-dose data. The scaled peak-dose curve agreed well with the valley-dose curve at larger distances from the peaks, but substantial deviations were observed at distances below ~ 81 μm from the peak center. This effect defines the spatial limit for reliable biological dosimetry of valley doses and demonstrates the importance of direct valley-dose measurements in MRT research.

#### Dosimetry with nucleoplasmic bridges for peak and valley regions after MRT

**Fig. 2:**
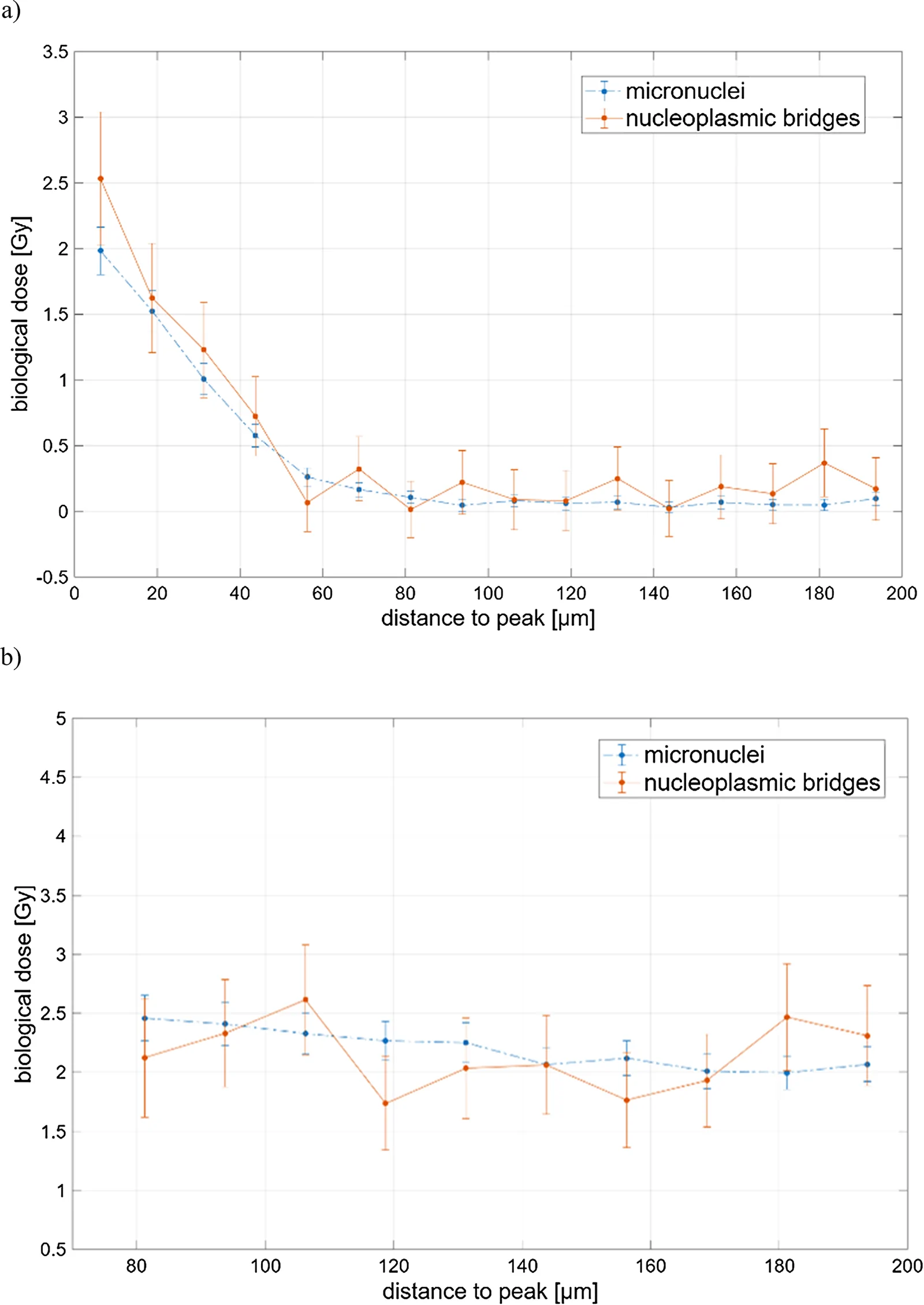
Biological dose reconstruction based on NPB frequency following MRT with **(a)** a peak dose of 2 Gy and a valley dose of 0.05 Gy and **(b)** a valley dose of 2 Gy. Cells were scored relative to the nearest peak center in bins of 12.5 μm. The biological dose reconstructed from the frequency of NPBs is shown in orange as a function of the distance from the peak center after MRT. For comparison, biodosimetry using micronuclei is shown as a blue dotted line as published by [36]. Each data point represents the mean ± standard deviation; more than 1,000 cells were analyzed per data point, *n* = 3

For microbeam irradiation of CHO-K1 cell monolayers with a nominal physical peak dose of 2 Gy and a valley dose of 0.05 Gy, biological dosimetry based on nucleoplasmic bridges between daughter nuclei (Fig. 1D, E) due to misrepair, yielded an estimated biological dose of 2.53 ± 0.51 Gy (corresponding to 0.18 ± 0.03 NPB per binucleated cell) in the peak regions and 0.17 ± 0.24 Gy (0.06 ± 0.01 NPB per BNC) in the valley. When MRT was performed with a physical valley dose of 2 Gy and a corresponding peak dose of 81.34 Gy, NPB-based dosimetry in the valley yielded a biological dose of 2.31 ± 0.42 Gy (0.17 ± 0.02 NPB per BNC). In this high-dose setting, however, dosimetry within the peaks and up to 81 μm from the peak center was not feasible, as the extreme radiation levels caused extensive cell killing and prevented the quantification of NPBs, mirroring the situation for MN formation. Notably, NPB-based dosimetry showed no significant differences compared to MN-based dosimetry across the conditions tested (Fig. 2).

At the cytogenetic endpoint level, MRT with a physical peak dose of 2 Gy induced MN in 53.9 ± 5.5% of binucleated cells, while 18.0 ± 5.8% exhibited nucleoplasmic bridges in these peak regions. Co-occurrence analysis revealed that 72.8 ± 4.1% of cells with NPB also contained MN, whereas 24.1 ± 6.3% of cells with MN also harbored NPB. A subset of 13.2 ± 4.9% of cells displayed both endpoints simultaneously.

In the valley regions after MRT with a physical valley dose of 2 Gy, 59.1 ± 4.0% of binucleated cells exhibited MN and 15.8 ± 2.6% showed NPB. Here, 84.2 ± 6.8% of cells with NPB also had MN, while 22.3 ± 1.4% of cells with MN also had NPB, and 13.2 ± 1.0% of cells showed both damage types concurrently.

Statistically significant differences were absent between the cytogenetic damage patterns in peak regions after 2 Gy peak irradiation and valley regions after 2 Gy valley irradiation. These results underscore the robustness of the analysis of NPBs as a complementary biomarker to MN in MRT biodosimetry, with both endpoints yielding consistent biological dose estimates. Moreover, the observed co-occurrence of MN and NPB within individual cells is consistent with the concept that both endpoints arise from radiation-induced chromosomal damage. However, because the present study did not formally test whether the observed overlap exceeded that expected under statistical independence, these data should not be interpreted as direct evidence of a shared mechanistic origin. The non-overlapping fractions further indicate that each endpoint captures complementary aspects of radiation-induced chromosomal instability.

### MRT-induced DSB damage in a 3D tissue model

#### MRT-induced DSB damage

Since radiation-induced bystander effects [25] cannot be fully represented in monolayer cultures, we next employed a reconstructed 3D human epidermis tissue models and irradiated these with microbeams. DNA double-strand break damage was subsequently analyzed by immunofluorescence staining of focal γH2AX and 53BP1 DSB-surrogate markers, followed by digital high-resolution spatial mapping of DSB foci distribution [27].

To specifically assess bystander responses, the number of DSB-associated foci in cells located in the MRT valley regions was compared to that observed after uniform broadbeam irradiation at an equivalent dose. This comparison provided a direct means to determine whether cells outside of the high-dose peak regions exhibited elevated DNA damage levels beyond those expected from the low physical valley dose alone, i.e., a bystander response.

For quantitative evaluation of DNA damage, two complementary parameters were analyzed for the DSB markers γH2AX and 53BP1: (i) the mean number of foci per cell (fpc), which reflects the average DSB burden per nucleus, and (ii) the percentage of cells carrying DSB foci among the total cell population (%cwf), which provides information on the fraction of cells affected by DSB damage. Together, these parameters allowed for a robust assessment of both, the intensity, prevalence and spatial distribution of radiation-induced DSB damage, thereby enabling the detection of subtle bystander effects in a physiologically relevant 3D tissue model.

Previous studies employing spatial DSB damage mapping in reconstructed epidermis models [27] suggested that radiation-induced bystander effects can be detected using this approach. As an initial step, DSB foci formation and repair were spatially analyzed across entire epidermal cross sections exposed to the different irradiation modalities and their evolution over time was quantified.

To assess the overall DSB damage burden, the total proportion of affected cells was calculated as the sum of all cells showing 53BP1 or γH2AX foci and colocalized 53BP1 + γH2AX foci. Across the full tissue, the mean percentage of DSB damage-carrying cells decreased from 63.1 ± 5.4% at 0.5 h post-irradiation to 26.0 ± 5.6% after 24 h, reflecting a progressive decline in the fraction of damaged cells consistent with ongoing DNA repair (Table 1).

**Table 1:** Overview of focal DSB damage at 0.5 and 24 h following MRT and broadbeam irradiation

time	MRT peak:19 Gy valley: 1 Gy	broadbeam dose: 1 Gy	control
**total %**			
0.5 h	63.1 +/- 5.4	54.8 +/- 3.4	5.4 +/- 1.1
24 h	26.0 +/- 5.6	25.2 +/- 0.9	6.1 +/- 0.5
**pan-γH2AX %**			
0.5 h	7.46 +/- 1.50	0.13 +/- 0.10	0.04 +/- 0.02
24 h	0.14 +/- 0.16	0.15 +/- 0.25	0.15 +/- 0.06
**53BP1**			
	% cwf		
0.5 h	59.0 +/- 5.7	54.0 +/- 3.8	5.06 +/- 1.18
24 h	25.5 +/- 5.5	24.3 +/- 1.4	5.05 +/- 0.20
	av. fpc		
0.5 h	1.92 +/- 0.27	1.45 +/- 0.17	0.07 +/- 0.01
24 h	0.41 +/- 0.11	0.31 +/- 0.02	0.08 +/- 0.02
**γH2AX**			
	% cwf		
0.5 h	2.64 +/- 0.07	1.35 +/- 0.89	0.39 +/- 0.08
24 h	0.44 +/- 0.24	1.00 +/- 0.61	0.98 +/- 0.44
	av. fpc		
0.5 h	0.034+/- 0.002	0.02 +/- 0.01	0.004 +/- 0.001
24 h	0.005+/- 0.003	0.013 +/- 0.009	0.012 +/- 0.007
**colocalized**			
	% cwf		
0.5 h	0.75 +/- 0.39	0.3 +/- 0.31	0.017 +/- 0.014
24 h	0.07 +/- 0.09	0.11 +/- 0.07	0.046 +/- 0.060
	av. fpc		
0.5 h	0.009+/- 0.005	0.003 +/- 0.003	0.0002 +/- 0.0002
24 h	0.0008 +/- 0.0011	0.0012 +/- 0.0008	0.0006 +/- 0.0008

Table 1:Overview of the average percentage of cells with DSB foci (% cwf) and average foci per cell (fpc) values over epidermis cross sections after MRT with 19 Gy peak dose and 1 Gy valley dose, and broadbeam irradiation with 1 Gy, and DSB damage in control samples, each assessed at 0.5 h and 24 h post-irradiation. ("Total %" refers to the average percentage of all cells showing at least one of 53BP1, γH2AX, co-localized 53BP1+γH2AX foci, or pan-γH2AX signal). Data are the average of n=6 sections from 2 independent epidermis models at 0.5 h post MRT, n=8 sections from 3 independent epidermis models 24h post MRT and n=9-13 sections from 3-4 independent models for sham- and broadbeam irradiation

**Fig. 3:**
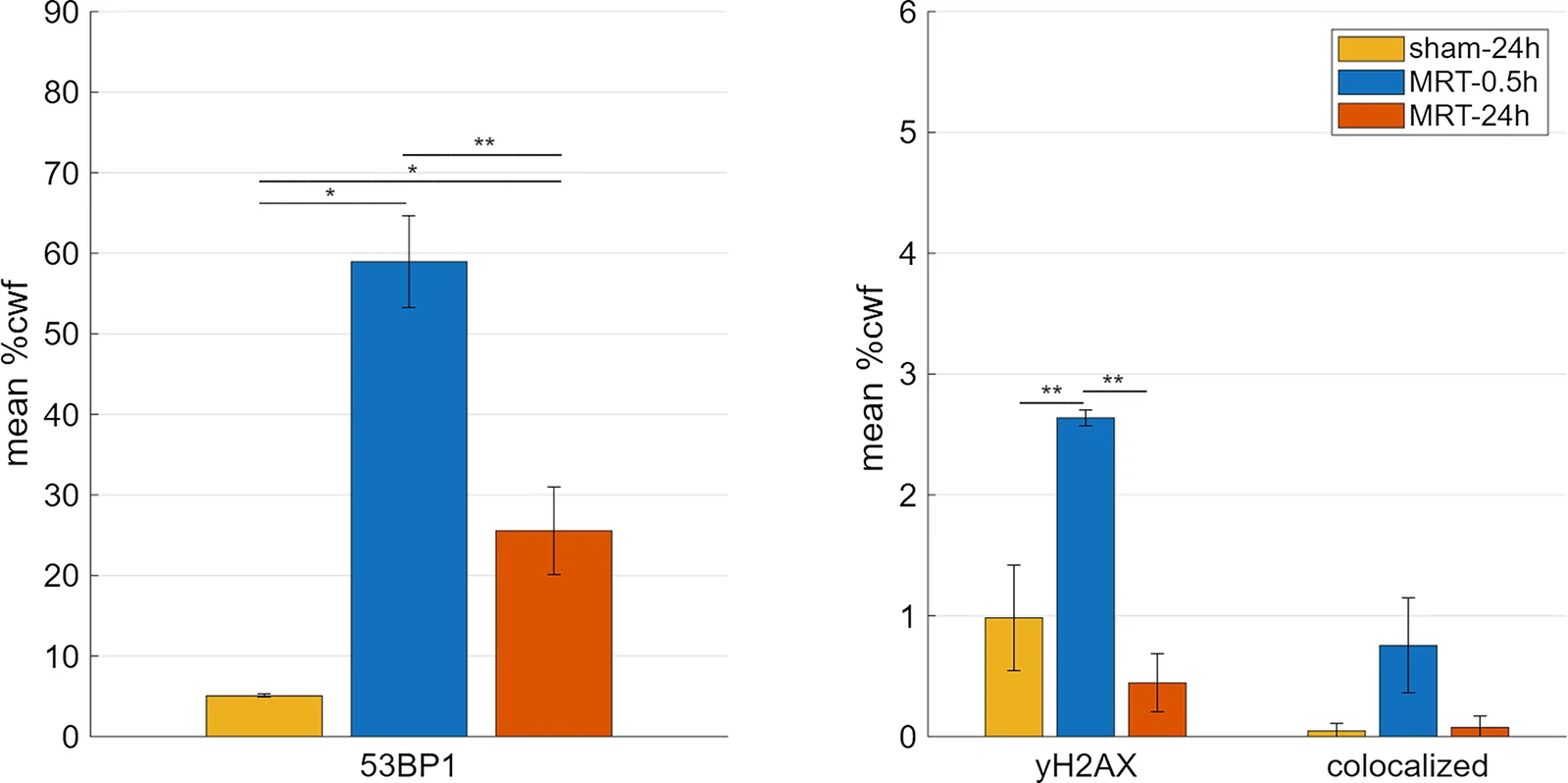
Average percentage of keratinocytes showing DSB damage foci (%cwf) at 0.5 h and 24 h after MRT (53BP1, γH2AX and co-localized foci) in epidermis models 0.5 h (blue) and 24 h (red) after microbeam irradiation. Sham-irradiated control samples at 24 h are shown in yellow, sham values for 0.5 h post irradiation are similar to 24 h (Table 1). It should be noted that terminally differentiated epidermis cells display more 53BP1 foci at lower doses (see text). Each data point represents the mean ± standard deviation of *n* = 6 sections from 2 independent epidermis models at 0.5 h post MRT and *n* = 8 sections from 3 independent epidermis models 24 h post MRT. In average we analyzed 2796 cells per section. * p-value < 0.05, ** p-value < 0.01

When analyzed separately, the percentage of cells with 53BP1 foci was markedly higher than for the other categories, decreasing from 59.0 ± 5.7% at 0.5 h to 25.5 ± 5.5% at 24 h. Importantly, even after 24 h the value remained significantly elevated compared to sham-irradiated controls (5.05 ± 0.20%; *p* < 0.01), indicating the persistence of residual DNA damage in a subset of cells (Fig. 3).

53BP1, a pro NHEJ factor [15], represented the most abundant DSB focus type in our models due to most cells being in G1/0 where NHEJ prevails. The average number of 53BP1 foci per cell declined from 1.9 ± 0.3 fpc at 0.5 h to 0.4 ± 0.1 fpc 24 h after irradiation, again consistent with incomplete repair after MRT irradiation (Table 1).

In contrast, γH2AX foci were less frequently observed in the epidermis models, with only 2.64 ± 0.07% of cells displaying γH2AX foci at 0.5 h post MRT, which dropped to 0.44 ± 0.24% at 24 h (*p* < 0.01). By 24 h, the frequency of cells with γH2AX foci was comparable to sham-irradiated levels (0.98 ± 0.44%) (Fig. 3).

**Fig. 4:**
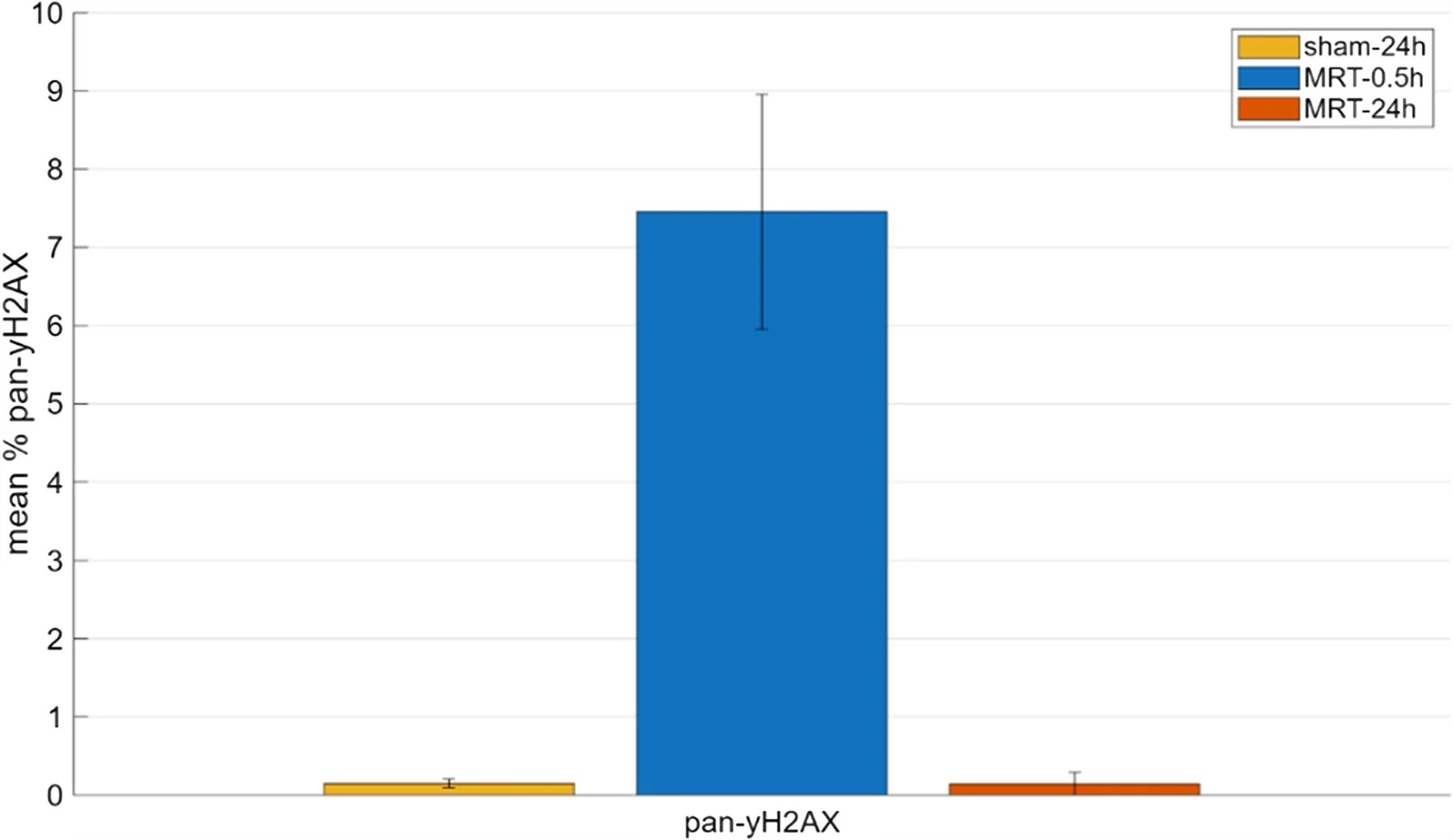
Average percentage of pan-γH2AX–positive cells (saturated γH2AX DNA damage response) across MRT-exposed epidermis models 0.5 h (blue) and 24 h (red) after irradiation. Sham-irradiated control samples at 24 h are shown in yellow, sham values for 0.5 h post irradiation are similar to 24 h (Table 1). Each data point represents the mean ± standard deviation of *n* = 6 sections from 2 independent epidermis models at 0.5 h post MRT and *n* = 8 sections from 3 independent epidermis models 24 h post MRT

In addition to discrete foci, we noted cells that displayed a pan-nuclear γH2AX staining pattern, resulting from extensive, genome-wide phosphorylation events associated with high acute dose-induced DSB damage in epidermis cells early after exposure [1, 27]. At 0.5 h post-MRT, 7.5 ± 1.5% of cells exhibited pan-γH2AX nuclei, whereas at 24 h this value had declined to 0.14 ± 0.16% (Fig. 4; Table 1). Although this reduction appears substantial, statistical comparison of the mean value per epidermis model did not yield significance (Welch’s t-test, *p* = 0.09), reflecting the small number of models that could be independently analyzed due to drop outs for technical reasons (*n* = 2 at 0.5 h; *n* = 3 at 24 h). By 24 h, the frequency of pan-γH2AX-positive cells was comparable to sham-irradiated controls (0.15 ± 0.06%), and no significant differences were detected between MRT and sham exposed tissues at this time point.

#### 3D distribution of DNA damage following MRT

**Fig. 5:**
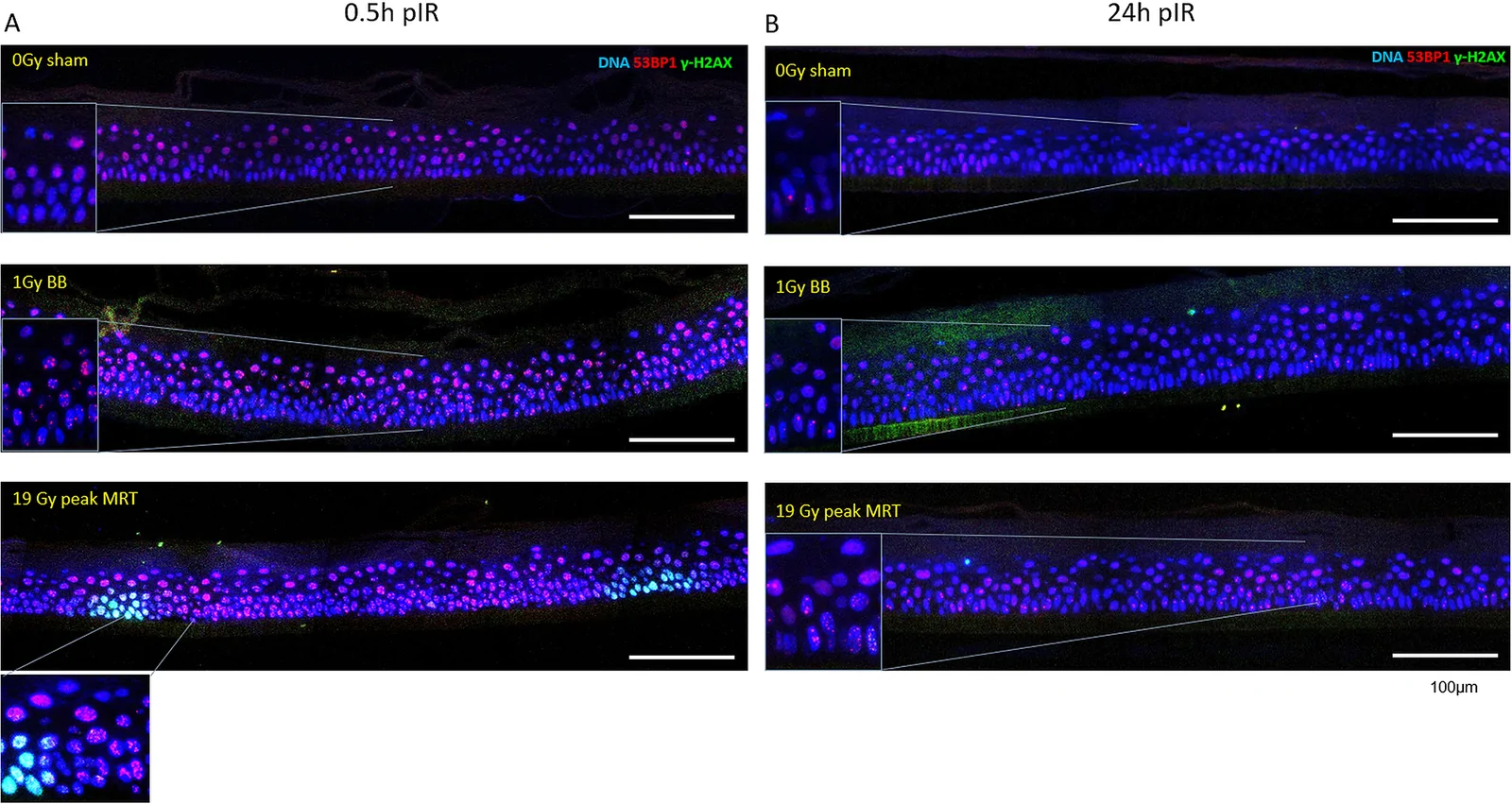
DNA damage pattern induced by BB and MRT at 0.5 h **(A)** and 24 h **(B)** post irradiation over the large part of typical epidermis tissue sections. Enlarged details are shown at the left margin of each image. Top row: sham irradiated models stained for DNA (blue, DAPI, nuclei) 53BP1 (red) and γ-H2AX (green), only few cells generally carry one 53BP1 focus. Center row: Broad beam irradiation induced numerous 53BP1 foci in epidermis nuclei that regressed in number with repair time (24 h, to the right). Lower row: MBRT irradiation-induced pan-γ-H2AX nuclei at the microbeam center (cluster of green nuclei to the left and right) with cells showing numerous 53BP1 and weaker γ-H2AX foci in the beam vicinity 0.5 h post irradiation. Most of the massive pan-γ-H2AX DNA damage was repaired 24 h after IR, leading to absence of pan-γ-H2AX nuclei, but cell nuclei still displayed several 53BP1 foci. For quantitative display of spatially mapped damage patterns at the single cell level see Fig. 6

**Fig. 6:**
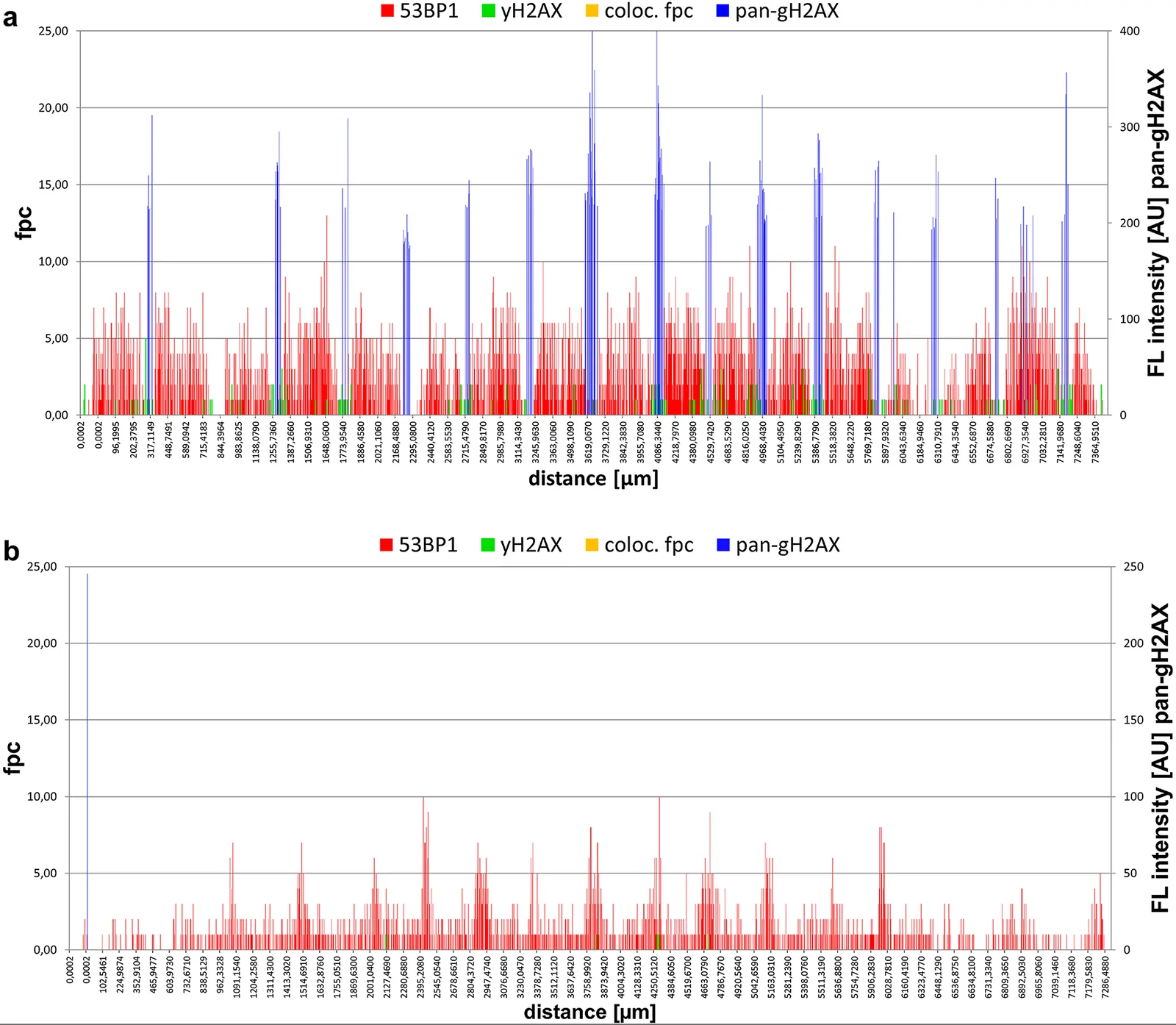
Spatial distribution of DNA damage in individual cells following MRT across an entire epidermis tissue section after MRT with a peak dose of 19 Gy and a valley dose of 1 Gy, **(a)** 0.5 h and **(b)** 24 h post IR. The positions of cells are mapped across a tissue section with corresponding DSB foci number per cell displayed as red (53BP1-fpc). Blue bars indicate the positions of cells with pan-γH2AX nuclei with bar height corresponding to fluorescence intensity (arbitrary intensity units, AU, Y axis to the right). It is evident that the 19 Gy dose in the MRT peaks leads to pan-nuclear γ-H2AX formation in the cell clusters within the MRT peaks (see Fig. 6a) 0.5 h post irradiation. Cells with elevated 53BP1 foci values are still seen 24 h post IR (Fig. 6b)

Next, we mapped the spatial distribution of the DNA damage types in individual cells across the epidermis models. Figure 5 shows the damage distribution induced in cross sections of epidermis models 0.5 h (Fig. 5A) and 24 h (Fig. 5B) after sham, BB and MRT irradiation. Figure 6 shows the spatially mapped distribution of the damage foci values of individual cells across sections 0.5 h (Fig. 6a) and 24 h (Fig. 6b) post MRT irradiation.

Pan-γ-H2AX nuclei showing a saturated γ-H2AX DNA damage response mapped at the 19 Gy dose peaks 0.5 h post irradiation, while the valley regions displayed cells with an average of three 53BP1 DSB foci per cell at this time point (Fig. 5A, lower row; 6a). At 24 h post irradiation, DNA repair progression was reflected by the absence of cell nuclei with a pan-γ-H2AX pattern (Fig. 5B, lower row), while the average 53BP1 fpc values in the valleys had dropped to 0.29, with cell nuclei with higher fpc values still marking the dose peaks (Fig. 6b). The average center-to-center distance between adjacent microbeam peaks was 430 ± 41 μm for all epidermal cross sections, consistent with the expected beam geometry.

At 0.5 h after MRT with a peak dose of 19 Gy and a valley dose of 1 Gy, clusters of pan-γH2AX–positive nuclei were observed at the positions corresponding to the microbeam peaks. The frequency with pan-γH2AX staining decreased progressively toward the valley regions (Fig. 6a), reflecting the steep dose gradients characteristic of MRT [36].

Cells displaying discrete γH2AX foci were also primarily located in the vicinity of the peaks in cells that did not show pan-nuclear γH2AX staining (Fig. 6a). This suggests that γH2AX foci preferentially marked localized DNA double-strand breaks in cells receiving lower doses at the peak margins, while pan-γH2AX indicates a confluent γ-H2AX signal covering the nucleus after massive DSB damage in cells directly traversed by the high dose peak of the beam, which is corroborated by DNA repair removing the pan-γ-H2AX signal in nuclei during the 24 h post irradiation. These results are corroborated by similar observations made after proton minibeam radiotherapy of epidermis models, where sharply localized clusters of pan-γ-H2AX nuclei at the ~ 25 Gy dose peaks were flanked by nuclei showing elevated γ-H2AX DSB foci numbers [27].

In contrast, 53BP1 foci followed a different spatial distribution. Cells harboring 53BP1-marked DNA damage were most frequently observed in the valley regions, with the frequency of positive cells decreasing toward the peak centers (Fig. 6a) 0.5 h post MRT. This pattern mirrors the one seen after proton MBRT [27] and is likely explained by the dilution of 53BP1 molecules to submicroscopic levels in nuclei exposed to extremely high peak doses [27], where the number of MRT-induced DSBs (~ 720 per nucleus at 18 Gy [37]) likely exceeds the capacity for efficient 53BP1 focus formation [9], thereby masking its detection in heavily damaged cells.

Interestingly, the proportion of cells positive for 53BP1 exceeded that of γH2AX-positive cells throughout the epidermis. A similar observation was made in our previous study using proton minibeam irradiation where it was noted that γ-H2AX formation was efficient only at minibeam doses > 4 Gy at the beam shoulders [27]. One possible explanation is that the terminally differentiated keratinocytes of the epidermis predominantly rely on non-homologous end joining for DSB repair, in which 53BP1 plays a central role [15] and that the ATM-dependent DDR requires higher doses for its activation ([27] and refs therein). Consequently, 53BP1 recruitment at DSBs is more readily detected than γH2AX in this epidermis tissue model.

**Fig. 7:**
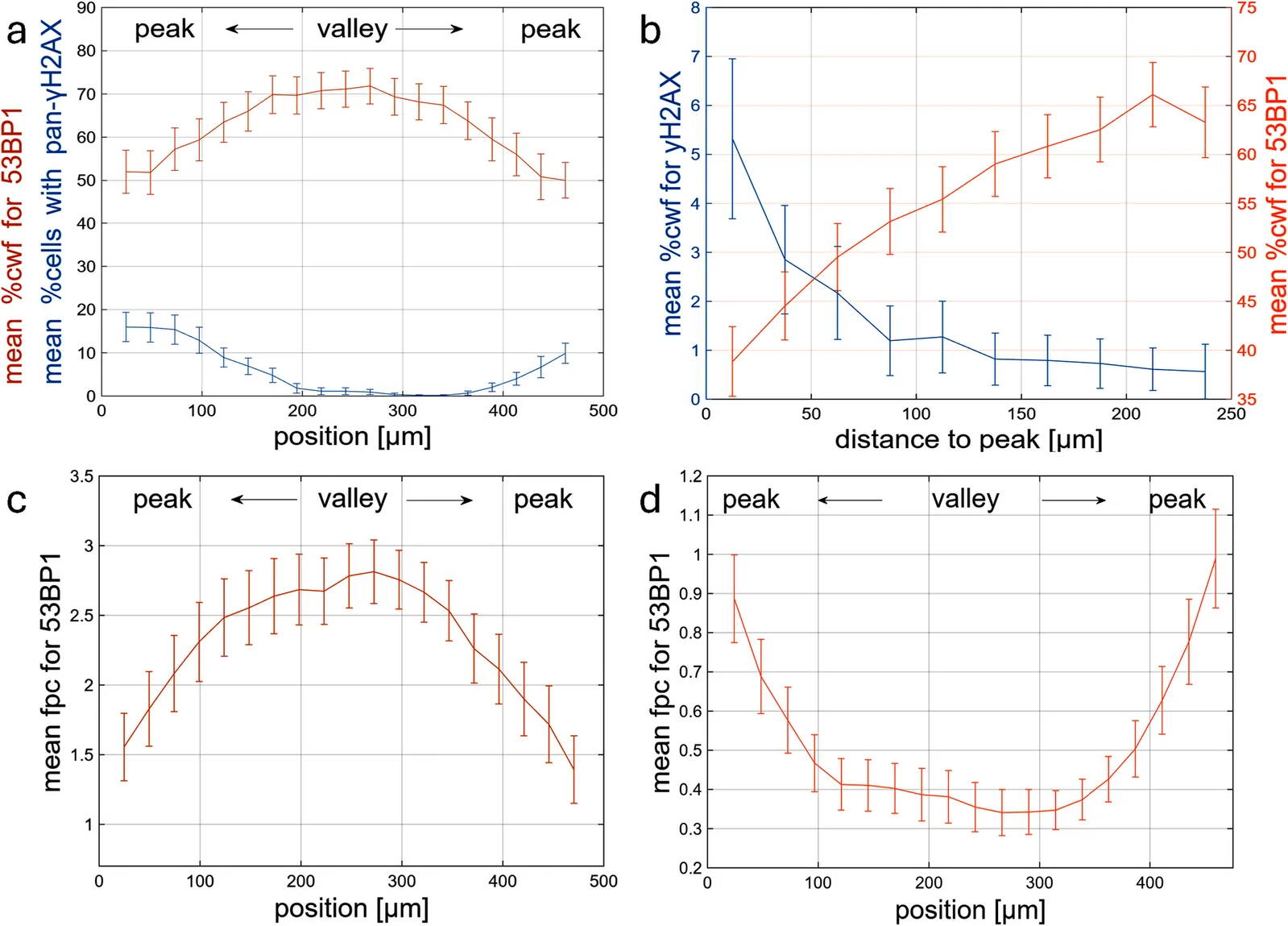
Spatial distribution of DNA damage 0.5 h **(a-c)** and 24 h **(d)** after MRT. **(a)** 53BP1 DSB foci/cell damage distribution across a valley region as revealed by the mean percentage of cells (%cwf) with ≥ one 53BP1 focus (orange) and the mean percentage of pan-γH2AX-positive cells (blue) in 25 μm bins across a microbeam peak-to-peak distance. **(b)** Percentage of cells with ≥ 1 γH2AX focus (blue) and 53BP1 DSB focus/cell (orange) as a function of distance from the peak center at the left margin. **(c)** Valley region showing the mean number of 53BP1 foci per cell (fpc) across one peak-to-peak distance. **(d)** Spatial distribution of 53BP1 foci in cells 24 h after MRT across a valley region of 100–350 μm showing the mean number of 53BP1-foci per cell peak-to-peak in a along a cross-section detail. Data indicate that high dose-exposed cells are left with elevated damage 24 h post MRT. For a–d, each data point represents the mean fpc ± standard deviation as derived in 25 μm bins from the nearest peak across all microbeam peak to peak distances within one epidermis tissue section. The values shown are from individual experiments. It is evident that the spatial positioning of pan-γ-H2AX nuclei and higher 53BP1 foci/cell values are inversely correlated 0.5 h post irradiation, meaning that 53BP1 fpc values are non-informative in high dose-exposed cells early after MRT (see text)

Quantitative analysis confirmed this spatial gradient. The mean proportion of cells with 53BP1 foci, averaged across all sections (%cwf), was significantly higher in the valleys (66.7%cwf ± 2.9) compared to the peak regions (42.2 ± 9.3%) (Table 2; Fig. 7a, b). A similar trend was observed for the mean number of foci per cell: 2.28 ± 0.25 fpc in the valleys versus 1.22 ± 0.32 in the peak dose regions (Fig. 7c) (*n* = 6 sections from 2 independent epidermis models). These findings indicate that, in the 3D epidermal context, 53BP1 provides greater sensitivity for detecting DSBs in low dose valley-exposed cells, whereas pan-γH2AX staining primarily captures massive DNA damage in high dose peak-irradiated cells. The apparent reduction in 53BP1 foci formation at the highest peak doses should therefore not be interpreted as a failure of the marker, but rather as a consequence of the biological limits of discrete focus formation under conditions of extensive DNA damage. In this respect, it has been noted that 53BP1 focus formation relies on a limited protein pool that may be diluted by excessive damage, as noted after sequential carbon ion irradiation [9].

Accordingly, 53BP1 is not a universal biomarker across the entire MRT dose range. Instead, its principal strength lies in the detection of discrete DNA double-strand breaks in the low and intermediate-dose range, particularly within the valley regions of MRT. In contrast, pan-γH2AX staining remains highly sensitive in high-dose irradiated peak regions, where widespread chromatin phosphorylation rather than distinct repair foci formation predominates. The complementary response pattern of both markers therefore provides a more complete characterization of spatial DNA damage after MRT than either marker alone.

At 24 h post-MRT, the spatial distribution of DNA damage in the 3D epidermal tissue models showed clear differences compared to the early time point (0.5 h). Clusters of 53BP1 foci-containing cells were observed at regular intervals corresponding to the microbeam geometry, marking the same peak dose positions that had been dominated by pan-γH2AX–positive cells shortly after irradiation. By this later time point, pan-γH2AX–positive nuclei were only sporadically detected and occurred at frequencies indistinguishable from sham-irradiated controls (Figs. 5b and 6b), indicating that cells initially exhibiting genome-wide phosphorylation due to massive DSB damage likely underwent repair, reflected by the absence of this damage pattern at 24 h. In agreement, radiation-induced apoptosis was shown to commence about 1 day after pMBRT [27]. Isolated cells with γH2AX foci persisted at 24 h and primarily localized to the peak regions (Fig. 6b).

Quantitative analysis of the 53BP1 DSB foci distribution revealed a distinct shift relative to the early post-irradiation time point. Whereas 0.5 h after MRT, 53BP1-marked damage was more prominent in valley-exposed cells, at 24 h the highest frequency of 53BP1 foci was observed in the peak regions, with the number of foci gradually decreasing toward the valleys (Fig. 7d). On average, 22.7 ± 4.2% of valley cells contained 53BP1 foci, compared with 30.0 ± 7.0% of peak cells. Similarly, the mean number of 53BP1 foci per cell was higher in the peaks (0.62 ± 0.19 fpc) than in the valleys (0.29 ± 0.07 fpc), based on *n* = 8 sections from 3 independent epidermis models.

#### Analysis of the valley region for bystander effects

To investigate potential bystander effects, focal DSB damage was specifically analyzed in the valley regions of epidermis models following MRT with a 1 Gy valley dose and compared to DNA damage induced by uniform broadbeam irradiation with the same physical dose. Since 53BP1 foci were the predominant and most reliable marker of the DNA damage response (DDR) in this epidermis tissue model, they were selected as the comparative endpoint. An overview of the results is presented in Fig. 6; Table 2.

**Fig. 8:**
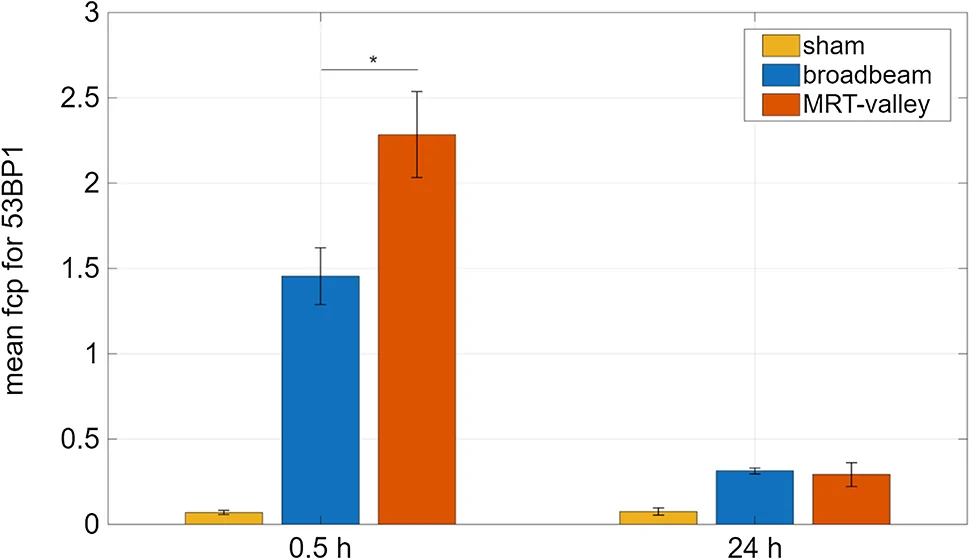
Comparison of 53BP1 DSB foci in the valley region after MRT and broadbeam irradiation. Number of 53BP1 foci per cell (fpc) after sham irradiation (yellowish), 1 Gy broadbeam irradiation (blue), and microbeam irradiation (dark orange) with a valley dose of 1 Gy and peak dose of 19 Gy (red), measured at 0.5 h (left) and 24 h (right) post-irradiation. For sham and broadbeam irradiation, the mean ± standard deviation was calculated over *n* = 9–13 sections from 3–4 independent epidermis models. For the MRT group, the mean ± standard deviation represents the average number of foci per cell in the valley region, calculated from *n* = 6–8 sections from 2–3 independent epidermis models. * *p* < 0.05

In sham-irradiated control tissues, only low background levels of DNA damage were detected. The mean number of 53BP1 foci per cell was 0.07 ± 0.01 fpc at 0.5 h and 0.08 ± 0.02 fpc at 24 h, with no significant difference between the two time points. The percentage of cells with foci remained consistently low at 5.1 ± 1.2% cwf at 0.5 h and 5.1 ± 0.2% cwf at 24 h (Table 2).

Broadbeam irradiation with 1 Gy induced a robust DDR, reflected by a significant increase in DNA damage at 0.5 h. The mean number of foci per cell rose from on average 0.07 ± 0.01 fpc in sham irradiated controls to 1.45 ± 0.17 fpc, with 54.0 ± 3.8% of cells harboring foci. At 24 h post-irradiation, damage values decreased but remained above background (0.31 ± 0.02 fpc; 24.3 ± 1.4% cwf).

MRT with a 1 Gy valley dose (corresponding to a peak dose of 19 Gy) induced a higher 53BP1 foci response in the valley regions at 0.5 h, with the mean number of foci per cell being 2.28 ± 0.25, with 66.7 ± 2.9% of cells displaying foci. Both values were significantly higher (*p* < 0.05) than those observed after broadbeam irradiation with the same nominal dose (Fig. 6; Table 2). At 24 h, however, the damage profile had converged: the average number of foci per cell was 0.29 ± 0.07 and 22.7 ± 4.2% of cells showed foci, closely matching the levels after broadbeam exposure. For both MRT and broadbeam irradiation, the average values for fcp at 24 h remained significantly above sham levels.

Taken together, these findings demonstrate that MRT initially induced a stronger 53BP1 response in valley-exposed cells compared to broadbeam irradiation with the same physical dose. This excess of radiation-induced 53BP1 foci at 0.5 h suggests the involvement of non-targeted effects, such as signaling from heavily irradiated peak regions into adjacent valleys, consistent with a radiation-induced bystander response. However, at 24 h post-irradiation, the focal 53BP1 DSB damage profiles in MRT valleys and broadbeam-irradiated tissues had largely converged, indicating that most bystander-associated excess damage was transient and repaired over time.

**Table 2:** 53BP1 foci/cell values in dose peaks and valleys 0.5 and 24 h post-MRT

Time	MRT-peak 19 Gy	MRT-valley 1 Gy	broadbeam 1 Gy	sham 0 Gy
	**% cells with 53BP1 foci**			
0.5 h	42.2 +/- 9.3	66.7 +/- 2.9	54.0 +/- 3.8	5.1+/-1.2
24 h	30 +/- 7	22.7 +/- 4.2	24.3 +/- 1.4	5.1 +/- 0.2
	**average 53BP1 foci per cell**			
0.5 h	1.22 +/- 0.32	2.28 +/- 0.25	1.45 +/- 0.17	0.07 +/- 0.01
24 h	0.62 +/- 0.19	0.29 +/- 0.07	0.31 +/- 0.02	0.08 +/- 0.02

Table 2: Values for percentage of cells with 53BP1 foci and average 53BP1 foci per cell after MRT shown for the peak region (19 Gy) and valley region (1 Gy), as well as after Broadbeam irradiation (1 Gy) and sham irradiation, each measured at 0.5 h and 24 h post-irradiation. Data are the average of n=6 sections from 2 independent epidermis models at 0.5 h post MRT, n=8 sections from 3 independent epidermis models 24h post MRT and n=9-13 sections from 3-4 independent models for sham- and broadbeam irradiation

## Discussion

Our study shows that in vitro biological dosimetry using the CBMN assay, alongside with immunohistochemical detection of the DNA damage response by γH2AX and 53BP1 DSB-indicating foci in epidermis model sections, represent suitable and reliable methods for spatial DNA damage mapping following MRT. Thereby it was possible to determine the microbeam-induced DNA damage distribution at and below the micrometer-scale, both in cell cultures and in three-dimensional tissue models. This integrated experimental approach enabled us to distinguish direct radiation-induced DNA damage from transient bystander-associated responses and therefore provides further insight into the biological effects of spatially fractionated X-ray irradiation.

MN and nucleoplasmic bridge (NPB) frequency data showed a linear dose-response relationship up to 4 Gy, enabling the generation of biological reference curves for conversion of MN and NPB frequency values to biological dose. The MN assay failed to be applicable at doses > 4 Gy, which is likely due to saturation effects and cell-cycle arrest that limited the detectability of MN, a notion that agrees with earlier reports [19, 28]. This limitation is inherent to cytogenetic biodosimetry rather than specific to MRT. Similar to the dicentric chromosome assay, the CBMN assay relies on the successful completion of one mitotic division after irradiation. At high doses, severely damaged cells undergo reproductive cell death or permanent cell-cycle arrest and therefore do not contribute to the pool of scorable binucleated cells [19]. Consequently, cytogenetic biodosimetry is most reliable within the low- to moderate-dose range and is primarily suited for biological dose reconstruction in the biologically relevant MRT valley regions rather than for estimating the extreme peak doses. Radiation-induced MN demonstrated greater sensitivity and reduced variability compared to the endpoint of nucleoplasmic bridges, thereby enabling more precise conversion of MN frequencies into biological dose.

Using the MN and NPB reference curves allowed to reconstruct the biological dose distribution across MRT peaks and valleys and showed a strong agreement with the physical dosimetry obtained from Gafchromic film. Peak width and center-to-center spacing were accurately reproduced in situ, although limited spatial resolution of the MN scoring bins introduced minor deviations. Separate dosimetry of peaks and valleys further demonstrated that independent characterization in regions with steep dose gradients improves overall accuracy.

The comparison with homogeneous broadbeam irradiation was based on the MRT valley dose rather than the physical mean dose. This approach is consistent with the concept of the equivalent uniform dose (EUD), which assumes that heterogeneous and homogeneous dose distributions are biologically equivalent if they produce the same biological effect [22]. In MRT, the biologically effective dose is considerably lower than the physical mean dose because cells exposed to the extreme peak doses contribute little to the surviving population. Consequently, valley-dose comparisons provide a more biologically meaningful reference for assessing DNA damage responses than comparisons based on the physical mean dose.

While NPB-based dosimetry values validated the overall dose profile, it displayed reduced signal height and greater variance relative to MN. Despite its limitations, dosimetry based on NPBs remains viable and beneficial, particularly because NPB scoring requires no additional sample processing and can be performed concurrently with MN analysis in the CBMN assay. However, due to the inherently low frequency of NPB-positive cells, spatial interpretation of NPB distributions after microbeam irradiation relies on prior identification of peak and valley regions using MN counts. Consequently, meaningful spatial resolution based on NPBs alone is only achievable when combined with the parallel analysis of MN. Nevertheless, NPBs provide complementary mechanistic information, as they reflect dicentric chromosome formation resulting from misrepaired DNA double-strand breaks [6].

Investigation of DSB DNA damage induced by MRT in 3D epidermis models revealed that the spatial pattern of DSB damage at 0.5 and 24-hours post-irradiation mirrored the underlying microbeam geometry, affirming that MRT-induced DNA damage can be effectively resolved in three-dimensional tissue, capturing dose heterogeneity at the micrometer scale beyond conventional cell culture models, as has previously been observed for pMBRT [27].

Together, these results suggest a dynamic redistribution of detectable DNA damage with increasing repair time. The disappearance of pan-γH2AX signals between 0.5 h and 24 h indicates clearance of cells with massive DNA damage likely by DSB repair [27]. In parallel, the relative enrichment of 53BP1 foci in peak-exposed cells at 24 h implies that the residual damage persisting at later stages is concentrated in regions of highest radiation exposure, consistent with the presence of complex or slowly repairable double-strand break damage. By contrast, valley regions, though initially marked by elevated 53BP1 damage, showed a greater decline over time, suggesting more efficient repair and/or less complex DNA damage in cells that experienced lower doses.

Unlike previous studies reporting bystander effects in 2D cell culture [8, 17], no bystander responses were detected in the valley regions of 2D cell cultures in the present study, either through micronucleus or nuclear bridges formation. Several mechanisms have been proposed for the induction of bystander effects, including direct intercellular contact and the release of cytokines and chemokines into the extracellular medium [7]. In confluent monolayer cultures, gap junction intercellular communication (GJIC) is considered a key contributor to radiation-induced bystander signaling ([18]; Prise and O’Sullivan, 2009). In the present study, however, CHO-K1 cells were intentionally seeded at low density to facilitate proliferation and automated CBMN scoring, resulting in minimal cell-cell contact and making GJIC unlikely. Under these conditions, bystander signaling would be expected to occur predominantly through medium-mediated intercellular communication (MMIC). In contrast, the reconstructed 3D epidermis preserves tissue architecture and extensive cell-cell contacts, allowing both GJIC and MMIC to contribute to non-targeted signaling. Since the present study was not designed to discriminate between these pathways, the relative contribution of each mechanism remains to be determined.

It should be noted that the CBMN assay and γH2AX/53BP1 immunofluorescence interrogate different stages of the radiation response. Whereas γH2AX and 53BP1 detect the rapid induction and repair of DNA double-strand breaks within hours after irradiation, micronuclei and nucleoplasmic bridges represent persistent chromosomal damage that becomes evident only after completion of mitosis. Consequently, transient bystander-induced DNA damage may be detected by γH2AX or 53BP1 without necessarily resulting in increased MN or NPB formation, if the lesions are efficiently and correctly repaired before cell division. Therefore, the two approaches provide complementary rather than directly comparable information on MRT-induced DNA damage and bystander responses.

The limitation of the current 2D cell model led us to study DNA damage distribution in a 3D human skin epidermis model. While MN analysis is limited to 2D tissue sections and requires cell culture, DSB damage foci distribution can be studied in great spatial detail at subcellular resolution in 3D tissue models [27]. The current analysis revealed bystander responses following microbeam irradiation by 53BP1 DSB foci analysis, as most of the cells in a epidermis are terminally differentiated and in G_0_/G_1_ phase and 53BP1 foci are a reliable marker in this cell cycle phase where NHEJ repair dominates [24]. At 0.5 h post-irradiation, cells located in the valley regions of the 3D tissue showed a significantly higher number of average 53BP1 foci per cell (fpc) compared to those in broadbeam irradiated control tissues receiving the same dose. Since broadbeam exposure yields a uniform dose across the tissue, foci levels in this condition serve as a baseline for directly induced damage at a given dose. The observation that valley-region cells in microbeam-irradiated tissue contained more 53BP1 foci suggests a non-targeted, bystander-type DNA damage response. Since the peak regions absorb higher doses in an irradiated tissue section, it can be expected that diffusible signals like ROS and RNS are induced in these regions [25] and diffuse through the cell connections transmitting diffusible damage signals from directly irradiated peak regions to adjacent tissue cells in the valley MRT regions.

The disparity of bystander responses in the 2D and 3D models used emphasizes the role of tissue architecture and intercellular communication in modulating radiation responses. Unlike a sparse monolayer, the stratified epidermal construct maintains an intact, layered cellular tissue structure and a contiguous 3D environment, enabling both direct cell communication through gap junctions and indirect signaling through diffusion of secreted factors across the tissue. This aligns with significant differences in radiobiological responses known for 2D and 3D cell culture models (e.g [10, 26]). The use of the same CHO-K1 cell line model as in our previous MN biodosimetry study was intentional, as it enabled a direct comparison between MN- and NPB-based biological dose reconstruction without introducing variability arising from cell line-specific differences in radiosensitivity or DNA damage responses. Future studies should investigate the applicability of NPB-based biodosimetry in additional cell lines with different genetic backgrounds and radiation sensitivities.

The present study has several limitations. Biological validation was performed in one established cell line and one reconstructed epidermis tissue. Although these models provide reproducible systems for evaluating biological dosimetry and spatial DNA damage mapping, future studies should investigate whether the observed patterns are conserved across additional cell types, tissue models and in vivo systems. Accordingly, the present work should be regarded as a proof-of-principle study in two well-characterized model systems rather than as a general methodological benchmark.

Overall, our findings, particularly in 3D tissue context, indicate that bystander effects may contribute to the biological response induced by microbeams. Further research will have to elucidate the underlying mechanisms and to determine the extent to which non-targeted effects influence the therapeutic and normal tissue outcomes of MRT.

Our findings should also be interpreted in the context of the well-established normal tissue-sparing effect of MRT observed in vivo. Numerous experimental studies have demonstrated reduced normal tissue toxicity following planar microbeam irradiation despite the delivery of extremely high peak doses. Importantly, the present study addresses a different biological question by investigating the spatial distribution of the early DNA damage response rather than long-term tissue outcome. The transient increase in DNA damage markers observed in MRT valley regions therefore does not contradict the established tissue-sparing effect. Instead, it indicates that early non-targeted DNA damage responses may occur in cells receiving low physical doses. Whether such damage ultimately persists depends on subsequent DNA repair, intercellular communication, tissue regeneration, vascular responses, and immune processes, many of which cannot be recapitulated in the simplified in vitro models used here. Consequently, our findings complement rather than challenge the current understanding of MRT by providing high-resolution insight into the early biological events that precede the long-term tissue responses observed in vivo.

## Conclusions

In conclusion, our study demonstrates that combining cytogenetic biodosimetry with spatially resolved DNA damage mapping in complementary 2D and 3D models provides a suitable approach for investigating the biological effects of MRT at cellular resolution in the two model systems investigated here. This integrated approach enables quantitative assessment of spatial dose deposition and biological responses in both cell cultures and tissue models. Using this approach, we identified transient bystander-associated DNA damage responses in MRT valley regions and persistent DNA damage in peak regions, thereby providing further insight into the early biological response to MRT. The presented methodology should facilitate future mechanistic studies and the biological optimization of MRT. Its applicability to other cell types, tissue models and in vivo systems remains to be established.

## Data Availability

All data generated or analysed during this study are included in this published article .

## Abbreviations

BNC: Binucleated cell
CBMN: Cytokinesis-block micronucleus
ctc: Center-to-center
%cwf: Percentage of cells with foci
DDR: DNA damage response
DSB: Double-strand break
fpc: Foci per cell
IF: Immunofluorescence
IQR: Interquartile range
NPB: Nucleoplasmic bridge
MN: Micronuclei
MRT: Microbeam radiation therapy
PBS: Phosphate-buffered saline
NHEJ: Non-homologous end joining
PFA: Paraformaldehyde
pMBRT: Proton minibeam radiotherapy
PVDR: Peak-to-valley dose ratio
SD: Standard deviation
